# Gaze Restriction and Reactivation of Place-bound Content Drive Eye Movements During Mental Imagery

**DOI:** 10.5334/joc.316

**Published:** 2023-08-31

**Authors:** Lilla M. Gurtner, Walter F. Bischof, Fred W. Mast

**Affiliations:** 1 Department of Psychology, University of Bern, Bern, Switzerland; 2 Department of Psychology, University of British Columbia, Vancouver BC, Canada

**Keywords:** visual imagery, eye movement, looking at nothing, recurrence quantification analysis, individual differences

## Abstract

When we imagine a picture, we move our eyes even though the picture is physically not present. These eye movements provide information about the ongoing process of mental imagery. Eye movements unfold over time, and previous research has shown that the temporal gaze dynamics of eye movements in mental imagery have unique properties, which are unrelated to those in perception. In mental imagery, refixations of previously fixated locations happen more often and in a more systematic manner than in perception. The origin of these unique properties remains unclear. We tested how the temporal structure of eye movements is influenced by the complexity of the mental image. Participants briefly saw and then maintained a pattern stimulus, consisting of one (easy condition) to four black segments (most difficult condition). When maintaining a simple pattern in imagery, participants restricted their gaze to a narrow area, and for more complex stimuli, eye movements were more spread out to distant areas. At the same time, fewer refixations were made in imagery when the stimuli were complex. The results show that refixations depend on the imagined content. While fixations of stimulus-related areas reflect the so-called ‘looking at nothing’ effect, gaze restriction emphasizes differences between mental imagery and perception.

## Introduction

Eye movements during mental imagery carry information about processes that underlie mental imagery. For example, when a previously seen picture is imagined, fixation distributions resemble those from actually looking at the picture. This is known as the ‘looking at nothing’ effect. This spatial similarity has supported the notion of the spatial nature of mental images (Kosslyn, 1994) as opposed to mental images relying on a propositional code (Pylyshyn, 1981, 2002; for a review see J. Pearson & Kosslyn, 2015). Furthermore, a strong spatial similarity of eye fixation distributions in perception and mental imagery is beneficial for later retrieval of the imagined content (Bochynska & Laeng, 2015; Johansson & Johansson, 2014; Jordana S. Wynn, Olsen, Binns, Buchsbaum, & Ryan, 2018). This suggests that ‘looking at nothing’ has functional value. Over all, the ‘looking at nothing’ effect is a consistent and reliable finding. However, it depends on how fine-grained the spatial analysis of fixations is. In a recent paper (Gurtner, Bischof, & Mast, 2019), we showed that the ‘looking at nothing’ effect is present when comparing the number of fixations in quadrants, whereas the underlying scan paths in imagery and perception show few similarities, as analyzed with MultiMatch (Dewhurst et al., 2012) and ScanMatch (Cristino, Mathôt, Theeuwes, & Gilchrist, 2010).^1^ Thus, the current understanding of the spatial characteristics of eye movements in mental imagery is still incomplete. More importantly though, little attention has been devoted to properties of eye movements in mental imagery other than their spatial similarity with eye movements in perception.

Indeed, eye movements in imagery have other unique characteristics. Typically, fixations in mental imagery have a smaller spread (Brandt & Stark, 1997; Gurtner et al., 2019; Johansson, Holsanova, & Holmqvist, 2006) and last longer (Brandt & Stark, 1997; Recarte & Nunes, 2000). Another dimension of eye movements is their temporal dynamics, that is, how eye movements evolve over time. The temporal dynamics are of special interest for imagery research for at least three reasons. First, the temporal dynamics provide descriptive information about eye movements in imagery themselves, without directly relating them to eye movements made in perception Dewhurst et al. (2012). Second, in two recent studies, we showed that eye movements in imagery differ in terms of their temporal dynamics from those made during perception of the same information (Gurtner et al., 2019; Gurtner, Hartmann, & Mast, 2021). For example, in imagery, participants made more and more systematic refixations and the refixations happened sooner in time. The origin of these differences remains unclear. Third and most importantly, temporal dynamics of eye movements allow to test hypotheses about the maintenance of mental images, because, as we will show, different processes manifest themselves differently in temporal gaze dynamics. Here, we focus on the imagery maintenance processes responsible for refixations during imagery.

There are at least two different possible mechanisms that can lead to refixations in imagery. On the one hand, refixations can occur because the mental images tend to fade rapidly (De Beni, Pazzaglia, & Gardini, 2007; Farah, 1989; Kosslyn, 1994) and the place-bound content needs to be reactivated by refixations to the relevant areas (Ferreira, Apel, & Henderson, 2008; Foerster, 2018; Laeng & Teodorescu, 2002; Mast & Kosslyn, 2002; Scholz, Klichowicz, & Krems, 2018; Jordana S. Wynn et al., 2016). This is in line with findings suggesting that eye movements serve the maintenance of imagined content (Damiano & Walther, 2019; D. G. Pearson, Ball, & Smith, 2014).

On the other hand, refixations in mental imagery can also arise because participants restrict their gaze in mental imagery, as illustrated by the lower spread and the reduced number of fixations in mental imagery (Brandt & Stark, 1997; Johansson et al., 2006). In particular, generating and maintaining mental images requires cognitive resources and therefore, it is particularly cost-sensitive, which in turn influences how many eye movements will be planned and executed. Restricting eye movements could be a means to avoid interference from unavoidable perceptual input when eyes are open. Such input can influence (J. Pearson, Clifford, & Tong, 2008; Teng & Kravitz, 2019) and even interfere with mental imagery (Wais, Rubens, Boccanfuso, & Gazzaley, 2010). Protection against perceptual input by restricting the gaze to one particular area can thus also be a means to maintain imagined visual content. Such a restriction of the gaze would naturally also lead to increased refixations. In summary, refixations can arise from reactivation of place-bound content or from restriction of the gaze.

The goal of the present experiment is to distinguish between these two possible mechanisms of imagery maintenance by means of their temporal dynamics. This requires the analysis of the temporal gaze pattern leading to a more complete picture of the underlying mechanisms. Before we elaborate how eye movement characteristics can disambiguate between reactivation of place-bound content or gaze restriction, we provide more details on how we quantify temporal gaze dynamics.

Temporal gaze dynamics can be analyzed by recurrence quantification analysis (RQA). RQA is commonly applied to the analysis of dynamic nonlinear systems that change over time. For example, it has been used to describe recurring patterns in changing water level of the sea (Webber Jr & Zbilut, 2005) or to the breathing patterns of anesthetized patients (Webber Jr & Zbilut, 1994). It has also been successfully applied to the analysis of eye movements in visual perception (Anderson, Bischof, Laidlaw, Risko, & Kingstone, 2013; Bischof, Anderson, & Kingstone, 2019; Farnand, Vaidyanathan, & Pelz, 2016; Vaidyanathan, Pelz, Alm, Shi, & Haake, 2014): it computes the percentage of recurrent fixations, or refixations of previously inspected areas in a single scan path. Recurrent fixations are fixations that are closer to one another with respect to a predefined threshold, usually the area covered by the fovea (Anderson et al., 2013; Zhang, 2020). To our knowledge, we were the first to apply this method to eye movements during mental imagery (Gurtner et al., 2019, 2021). In this context, three RQA measures provide particularly important information: First, the percentage of recurrent fixations indicates how often an observer refixates previously inspected areas. Second, the CORM value (Center of Recurrence Mass) describes the general timing of refixations. When refixations are temporally close, that is, when the gaze returns to an area after few or no fixations to other areas, the CORM value is low. Conversely, when there are many intermediate fixations to other parts of a stimulus between refixations of an area, the CORM value is high (see also Anderson et al., 2013). Third, the determinism value indicates the extent to which recurrent fixations follow the same sequence of previous fixations. It reflects how systematic or repetitive a sequence of refixations is (Anderson et al., 2013). In previous research, we showed that the imagined content influences RQA measures (Gurtner et al., 2019, 2021). In the present work, we investigate the relevant dimensions further by varying the complexity of mental images. The complexity allows to determine whether refixations in imagery originate from reactivation of place-bound content or whether they originate from gaze restriction.

Specifically, if refixations during mental imagery arise because fading away of the mental images needs to be counteracted, recurrence should increase with image complexity: The more complex an image, the more demands it makes on the cognitive processes of the maintaining individual, and the less this individual can afford to make image-unrelated, intermittent fixations. Complex images also contain more distinct parts or segments, which would have to be visited and reactivated before returning to one of the previous parts, leading to a larger spread of fixations (at least if the different segments are spread apart too), and later refixations whereby CORM values increase.

Conversely, if refixations arise from restricting the gaze, participants would increasingly avoid making eye movements as stimuli become more difficult to maintain, reducing the spread of fixations, increasing the rate of refixations, and a diminished spatial relation to the original stimulus. Restricting one’s gaze would also increase determinism values when imagining complex mental images, because the systematicity or repetitiveness of eye movement patterns would increase. Likewise, it would lead to lower CORM values, as refixations tend to occur in closer temporal proximity when the area is restricted.

In sum, *how* image complexity affects RQA measures can distinguish between the two distinct hypotheses about the origin of refixations in mental imagery and thus can shed light on processes responsible for image maintenance. Refixations can arise in mental imagery because place-bound, fading pictorial content is reactivated or because gaze is restricted to avoid interfering perceptual input. RQA is the tool to distinguish between these two different accounts. If refixations originate from reactivation, we expect increasing recurrence, spread and CORM with increasing stimulus complexity. If refixations originate from gaze restriction, we expect increasing recurrence and determinism, but CORM and spread to decrease with increasing complexity.

**Figure 1:**
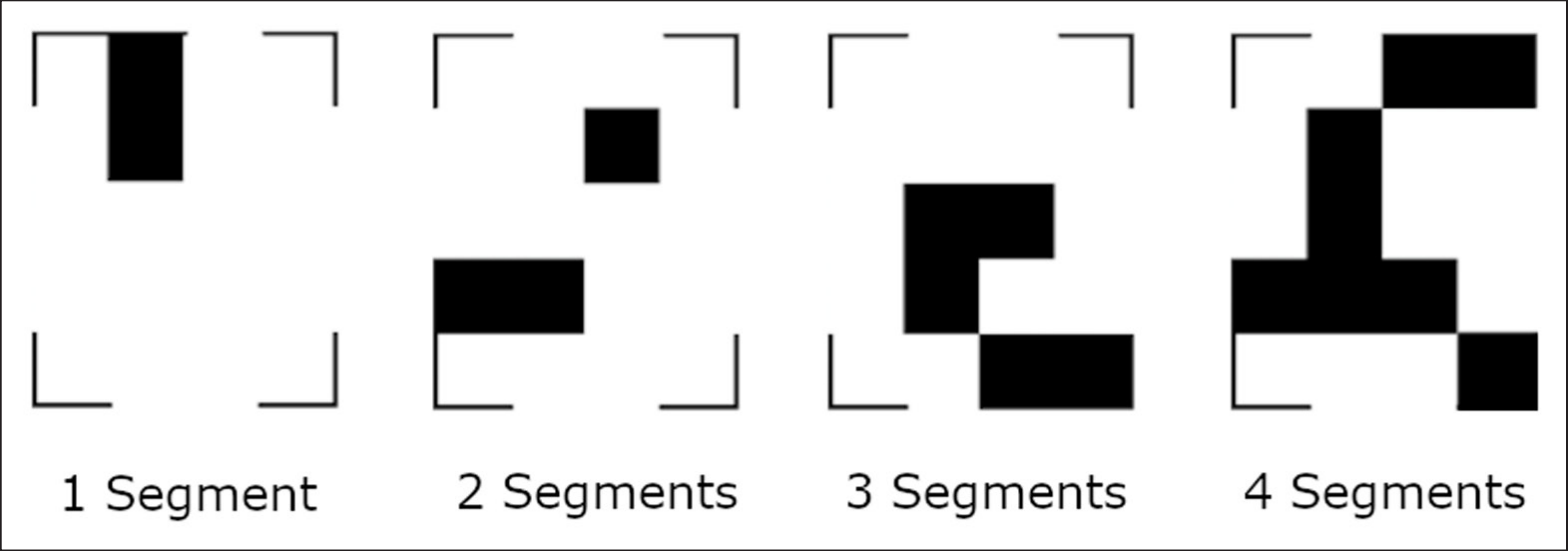
Examples for the stimuli of four different complexities. The segments were allowed to touch.

We investigated the origins of refixations and their dependency on image complexity in two experiments. In Experiment A, participants saw a geometric checkerboard pattern of varying complexity (Dror & Kosslyn, 1994, see Figure 1) for 700 ms and maintained it for 20 seconds, during which the screen remained blank. After the maintenance period, a cross was displayed, and participants had to indicate whether the cross would have fallen on a white or black part of the pattern. This task is, in parts, similar to tasks used in visuo-spatial working memory experiments (Schurgin, 2018; Sperling, 1963), where the analysis of eye movements has been key to understand visual continuity in active vision (Hollingworth, Richard, & Luck, 2008; Schurgin, 2018; Stigchel & Hollingworth, 2018). However, the task we used in this experiment differs from classical visual short-term memory tasks in that our participants maintained the stimulus for 20 seconds. Never the less, the proximity to visual working memory tasks and the fact that there is an argument that visual working memory and imagery might be different terms for the same phenomenon (Tong, 2013), inspired us to use dynamic visual noise (DVN) between the pattern and the blank screen in half of the trials (Dean, Dewhurst, Morris, & Whittaker, 2005; Dean, Dewhurst, & Whittaker, 2008; Dent, 2010; McConnell & Quinn, 2004; Orme, 2009), and to include a short-term visuo-spatial memory task (Visual Pattern Test). DVN was expected to interfere with the mental representation of the stimulus, which in turn should influence RQA parameters and thus impair mental image inspection at the end of the task. The main focus was to investigate whether the number of segments in the checkerboard pattern influenced the temporal gaze dynamics while participants maintained the pattern in imagery.

## Methods

### Participants

We recruited a total of 42 undergraduate students from the University Bern for the two experiments. They received course credit points as compensation for their participation. All participants had normal or corrected to normal vision. In the informed consent, we declared that the study was investigating whether pupil dilation can indicate general cognitive effort, i.e. whether individuals with high cognitive abilities show a smaller increase in pupil dilation when solving a memory task. The study was approved by the Ethics committee of the Faculty of Human Sciences of the University of Bern.

### Material

In both experiments, eye movements were registered using the EyeLink 1000 Software, which was provided by SR Research GmbH (Canada). The samples were recorded at 500 Hz. Using an infrared light sensitive camera, the eye tracking device inferred the gaze position from the corneal reflex and the pupil location. At the beginning of the experiment, we presented a 9 point grid for calibration. The participant rested their chin on a head support and faced the 26-inch display (51 × 28 cm, resolution: 1920 × 1080 pixel). The distance between the eyes and the display was approximately 76 cm. For the stimulus, we used a 4 × 5 grid where each square measured 109 × 108 pixels. Together with the square brackets, measuring each 12 pixels in width, the stimulus was presented centrally and measured 462 × 566 pixels (approximately 14.1 × 15.1 cm, 10 × 12.20 degrees of visual angle).

### Procedure

Both experiments comprised three parts: a maintenance task during which we measured participants’ eye movements, a paper-pencil test of visual short-term memory, and questionnaires assessing imagery vividness and demographic data. The participants spent two hours in the laboratory and were free to take breaks upon completion of each part.

**Figure 2:**
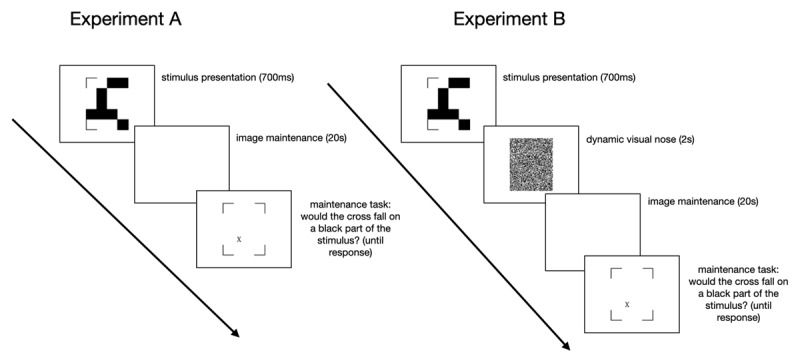
Timeline of the stimuli in the maintenance task.

The maintenance task began with the 9-point calibration, followed by 120 trials (see Figure 2). Each trial began with a drift correction, a fixation cross (for 500 ms) and a blank white screen (for 500 ms), after which participants saw a pattern of black blocks within a 4 × 5 grid (see Figure 1) for 700 ms. The patterns were constructed along the procedure of Dror and Kosslyn (1994). They were composed of straight segments containing one to four filled grid cells and were arranged in the 4 × 5 grid. The segments were allowed to touch and the stimulus also included the four corner brackets. The stimulus was followed by a blank screen that lasted for 20 seconds. Participants had to imagine the pattern they just saw while keeping their eyes on the screen. At the end of each trial, the four corner brackets reappeared and participants had to judge whether a cross on the screen would fall on a black or white background if the stimulus were still present. They indicated their answer by pressing one of two buttons on a keyboard in front of them. Finally, participants rated on a 7-point Likert-scale, how well they were able to maintain the stimulus. Experiment B was identical to Experiment A, except that we introduced two seconds of dynamic visual noise (DVN) between the stimulus and the blank screen in half of the trials. The other half of the trials was identical to all the trials of Experiment A. In previous research, participants imagined the stimuli for 15 seconds (Gurtner et al., 2019, 2021). Here, we chose a 20 second maintenance phase to ensure that a sufficient number of eye movements were made to compute RQA parameters. Moreover, a pretest with a 15 second maintenance phase has shown high accuracy in this task. A longer duration will likely increase difficulty and counteract a possible ceiling effect.

For the test of visual short-term memory, we used the Visual Pattern Test (VPT) (Della Sala, Gray, Baddeley, & Wilson, 1997), where participants looked at paper cards displaying a grid with an increasing number of squares, some of which were black and others white. The grids progressed in size from the smallest, a 2 × 2 matrix (with two black squares), to the largest, a 5 × 6 matrix (with 15 black squares). Each card was presented to the participant for three seconds. Immediately after viewing the card, the participants were required to mark the black squares on a response sheet. There was no time limit for responding, and participants could correct their responses if they wished. There were three patterns at each difficulty level and the test was terminated when the participant made a mistake in one of the three patterns at a given level of complexity. The number of correctly marked patterns was used to measure participants’ visuo-spatial short-term memory. At the end of the experiment, we asked participants what they thought the aim of the experiment was. No participant guessed that we were interested in eye movements during mental imagery.

### Dynamic visual noise

For Experiment B, we created the DVN in accordance with the procedures used in the literature. To optimize interference with the stimulus, we chose a short stimulus presentation time (Dent, 2010; Kemps & Andrade, 2012; Zimmer & Speiser, 2002), no inter-stimulus-interval (Borst, Kosslyn, & Denis, 2006), and a high rate of change of the DVN (Dean et al., 2005; McConnell & Quinn, 2004). The size of the noise was matched to the stimulus size (464 × 568 pixels). It was comprised of 116 by 142 cells of 4 × 4 black and white pixels. The monitor we used to present the stimuli had a frame rate of 144 Hz. We changed the noise-picture every fourth frame, leading to 36 changes per second. We decided on a rate of change of 50% since this rate has been found to exert a strong interfering effect on memory (Dean et al., 2005). Thus, in the 36 changing noise pictures per second, we wanted 50% of the cells (that is 8236 cells) to change color. Therefore, we constructed the noise pictures such that in each picture, 228 random cells changed color with respect to the previous one. The code used to generate the DVN can be found at: https://osf.io/h4tjc.

### Data analysis

#### Bayesian generalized regression models

We predicted the temporal gaze dynamics and the spread of fixations by the number of segments of our stimuli. To this end, we used Bayesian generalized hierarchical regressions. In a Bayesian analysis, a prior expectation about an effect is combined with empirical data, which results in a posterior estimate of the effect. Crucially, the posterior estimate is a distribution. In addition to the mean or median of the posterior, the distribution allows to quantify the certainty of the estimation through the range of the distribution. If the 95% density interval of this distribution (the credibility interval, CI) includes zero, the effect is typically absent. Conversely, if zero is not included in the CI, the effect is typically present. We used generalized regressions because they allow a linear model, which is restricted to normal data, to fit data that is not normally distributed. This is achieved by using a link function. In our case, we used a zero-one-inflated-beta distribution to fit the RQA measures (which vary between 0 and 100), and we used an ex-Gaussian distribution to fit the spread of fixations (which cannot be smaller than zero). The hierarchical structure of our regressions allows to model participant- and stimulus-specific variance. Thus, our models account for the fact that some participants have high recurrence values in mental imagery and others do not, and the fact that the influence of the number of segments in a stimulus can vary between participants. To this end, we included random intercepts and slopes for participants. Our model also accounts for the fact that some stimuli might be easier to imagine than their number of segments would actually predict. Therefore, we included random intercepts for stimuli. See Supplementary Material Section 1, for the model specifications of all regression models.

#### Implementation details

The fixations were defined by the Data Viewer software of SR research. We used the default settings for defining fixations: Each measurement is classified as part of a fixation if the pupil is visible to the eye tracker, the velocity of the gaze position does not exceed 30°/s and if its acceleration does not exceed 8000°/s. After extracting the fixations, we analyzed our data with R (R Core Team, 2015) and R-Studio (RStudio_Team, 2016) and with the packages of tidyverse (Wickham, 2017) to prepare and plot our data. We deleted all fixations that were either outside the screen, shorter than 100 ms or longer than 5 seconds. With these constraints in place, we included 90.30% of fixations in Experiment A, and 84.60% of fixations in Experiment B. To calculate the spread of fixations, we computed the average fixation point for each trial of each participant. Next, we calculated the distances of all fixations in the trial from the average fixation point. The median of these distances is reported as the spread of fixations. To compute the recurrence values, we used MATLAB and the functions made available by Anderson et al. (2013). Finally, the brms package (Bürkner, 2018) was used for the Bayesian generalized hierarchical regressions. To sample from the posterior distribution, we used four chains with 5000 iterations each. Once the regressions were run, we assessed model fit by checking the posterior predictive distributions. Based on the posterior, new data distributions are simulated. If a model fits the data well, the simulated data will resemble the actual empirical data. This was the case in all our models. The manuscript was produced using the papaja package (Aust & Barth, 2020). The data, study materials and analysis code will be made available at the Open Science Framework. This study was not preregistered.

## Results

Experiment A investigated the impact of image complexity on eye movements in mental imagery. Specifically, we tested whether the number of refixations (recurrence), their systematicity (determinism), and their timing (CORM) depended on the number of segments in a stimulus that was imagined by participants. In Experiment B, we added DVN to interfere with the mental representation of the stimulus and to make the task more difficult.

### Data quality and manipulation control

**Figure 3:**
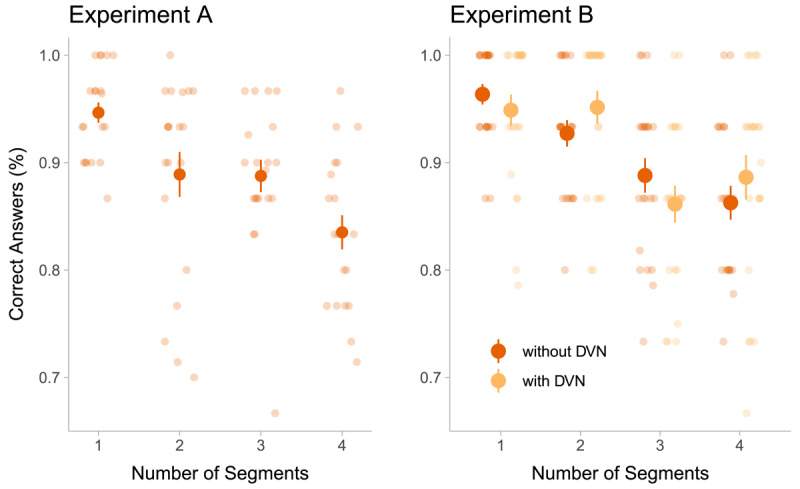
Performance in the maintenance task in Experiment A and B as a function of the number of segments in a stimulus. Opaque points indicate the mean across participants, and error bars indicate the standard error of the mean. In Experiment B, two seconds of dynamic visual noise (DVN) between the stimulus and the blank screen were introduced in half of the trials. The trials without DVN in Experiment B were identical to the trials in Experiment A. Transparent points show the average performance of individual participants over trials with the respective number of segments. Given that each participant absolved 120 trials in total, the points in the figure of Experiment A represent the average of 30 trials (120 trials/4 levels of segments), while the points in Experiment B represent the average accuracy of 15 trials (120 trials/4 levels of segments/2 levels of DVN).

Before looking at the effect of the number of segments on RQA measures, we ensured that participants performed sufficiently well in the maintenance task and that the different segments had the desired effect on accuracy. Overall, participants were correct in 87.80% (SD = 0.33) of the trials in Experiment A and in 91.20% (SD = 0.28) in Experiment B, see also Figure 3.

Next, we assessed whether complex stimuli were harder to imagine. The number of segments in a stimulus reduced participants’ accuracy in both experiments (binomial regressions, Experiment A: beta = –0.45, CI: –0.74 – –0.17, Experiment B: beta = –0.66, CI: –1.17 – –0.18). This means, the more complex the stimulus was, the more difficult it was to imagine correctly.

In Experiment B, the dynamic visual noise did not change the probability of a correct answer being given (beta coefficient of DVN = –0.31, CI: –1.28 – 0.67) and it did not change the impact of number of segments on accuracy (beta coefficient of the interaction DVN with number of segments = 0.13, CI: –0.18 – 0.45). Furthermore, model comparisons showed that including the DVN as predictor did not add to the predictive power of the model (elpd difference: –2.14, standard error of the difference: 1.51).

Taken together, these results show that the participants complied with the instructions. The number of segments had the desired effect of increasing the task difficulty of the maintenance task. This means that our procedure manipulated the maintenance process as intended and thus, differences in eye movements in the different complexity conditions can inform us about mental image maintenance processes.

### Image complexity and temporal gaze dynamics

**Figure 4:**
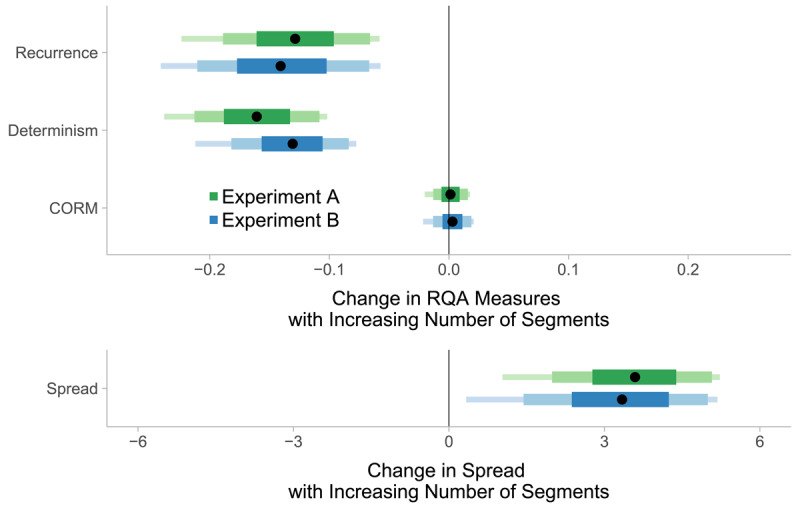
Estimated posterior distributions of the effect of number of segments on recurrence, determinism, CORM, and on the spread of fixations. The distributions indicate how the gaze dynamics and fixation spread change as the number of segments increases by one. Recurrence and determinism decrease and the spread of fixations increases as the number of segments increases, whereas the CORM value remains unaltered. The segments of the bars represent 90%, 80% and 50% of the posterior distributions and the black points represent their median.

Given that stimulus complexity increased task difficulty, we analyzed the influence of stimulus complexity on RQA measures to gain insight into the underlying maintenance mechanisms. We only analyzed trials in which we were certain that there was sufficient engagement with the maintenance task and in which imagery was successful. Therefore, we first excluded trials in which participants provided wrong answers. Second, we excluded trials in which participants found it difficult to maintain the stimulus, i.e. when the difficulty of maintenance rating was 1 or 2 out of 7 (18.80% in Experiment A, 15% in Experiment B, comparable amount of deletions for the different stimulus categories, see Supplementary Material, section 2). The number of segments of the stimulus influenced participants’ RQA measures. In both experiments, an increase in segments lead to a decrease in recurrence and determinism (see Figure 4). Thus, the more segments a stimulus had, the fewer refixations were made (i.e. the more fixations were made to previously unfixated areas) and the less systematic the remaining refixations became.

There are two possible confounds for this effect, both of which can be ruled out. First, the effects are independent of the spread of fixations, which was statistically controlled for: We included the spread of fixations as a predictor into the regressions. A second potential confound is the spread of the stimuli themselves. The screen was empty when participants maintained the stimuli. Nevertheless, eye movements in imagery could reflect the spread of the stimuli rather than reflecting the stimulus’ complexity. This would be expected, if eye movements predominantly reflect the ‘looking at nothing’ effect. To address this confound, we investigated whether the distribution of black pixels in each stimulus (i.e. how far black pixels were spread out in the stimulus) altered the relationship between the number of segments and recurrence. We found that the pixels’ distribution did not predict recurrence by itself, and including it as a predictor in a regression did not alter the effect of segment numbers on recurrence or enhance model fit. Therefore, we conclude that the effect of segment number on recurrence is not confounded with the pixels’ distribution in the maintained stimulus. For more details, see the Supplementary Material, section 3.

The reported effects indicate that the effect of the number of segments on RQA parameters is independent of the distribution of fixations. The estimated posterior distributions for the CORM measure showed dependence on the number of segments; they were centered around zero in both experiments. This means that the number of segments did not influence the timing of refixations. Moreover, DVN did not influence gaze dynamics or the spread of fixations (see the Supplementary Material, section 4).

Participants adapted their temporal gaze patterns when the stimulus was more complex in that they made fewer and less systematic refixations (as measured by recurrence and determinism) and more fixations to previously uninspected areas. At the same time, the spread of fixations increased as the number of segments in a stimulus increased (see Figure 4). Thus, participants made larger eye movements when they maintained a complex mental image and they restricted their gaze in trials where they maintained a simple mental image. This is in line with previous research on the relationship between the spread of fixations and task difficulty (Johansson, Holsanova, & Holmqvist, 2011; Scholz, Mehlhorn, Bocklish, & Krems, 2011; Jordana S. Wynn et al., 2018).

**Figure 5:**
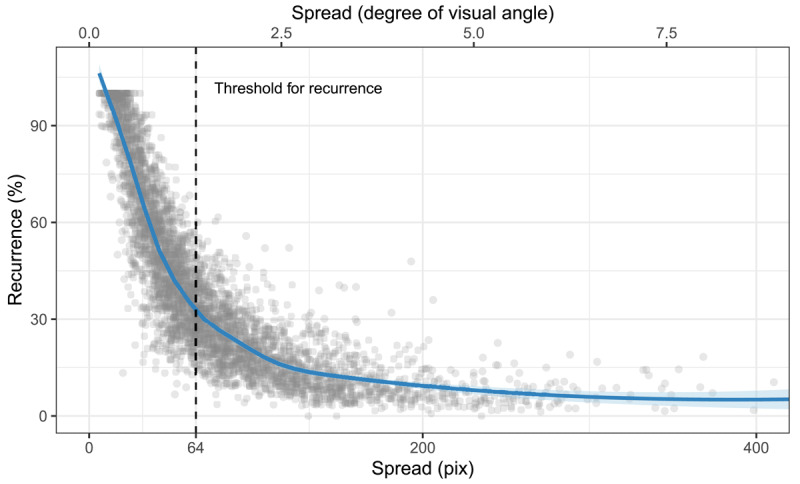
Relationship between the spread of fixations and recurrence. The dashed line shows the threshold distance between two fixations used to define refixations. Although theoretically possible, no trials show high recurrence and a high spread of fixations at the same time (no points are in the upper-right corner of the plot). Refixations in mental imagery happen predominantly in combination with low spread of fixations (gaze restriction). The plot has been truncated after 400.

Refixations can arise from both restricting the gaze or from making repeated fixations to *distal*, stimulus-related areas, *i.e. the spread of fixations does not necessarily relate to the rate of refixations*. In our results, refixation rates (recurrence) decreased as stimulus complexity increased, and at the same time, the spread of fixations increased. To understand the origin of this result, the relationship between the spread and recurrence, Figure 5, is paramount. The figure shows that trials with high recurrence rates (above 50%) have a low median spread of fixations (lower than the threshold distance to define recurrence, dashed line). Thus, refixations happen particularly often in trials with a low spread of fixations. Conversely, there was no trial in which participants moved their eyes considerably and, at the same time, revisited the same distant locations repeatedly (no trial falls into the upper-right corner of the graph). If refixations originated from re-activation of place-bound content, we would expect to find at least some trials (those with more widely-spread segments) in which participants show widely spread fixations and higher recurrence levels. Therefore, refixations mostly arise from restricting the gaze to a small area. It could be argued that the relationship between recurrence and the spread of fixations is caused by the specific spatial layout of our stimuli, as they did not cover the entire screen. However, a reanalysis of previous data (Gurtner et al., 2021), where stimuli covered the entire screen, showed that, in mental imagery, the same pattern is present and even consistent across different types of stimuli (art, faces and landscapes, see Supplementary Material, section 5). Taken together, refixations originate predominantly from gaze restriction. The process responsible for gaze restriction is attenuated as difficulty to maintain the mental image increases.

### Interindividual differences

In addition to our general findings, the analysis shows large inter-individual variance in the degree of refixations. In keeping with our previous results (Gurtner et al., 2019), some participants in the current experiment made few refixations (minimum of average recurrence, Experiment A: 9.08%, Experiment B: 8.87%) while others directed the vast majority of fixations to already inspected areas (maximum average recurrence, Experiment A: 72.70%, Experiment B: 95.40%). This was also reflected in the high standard deviations of the random intercepts between participants (Experiment A: 1.27, CI: 0.93 – 1.75; Experiment B: 1, CI: 0.72 – 1.40, please refer to the Supplementary Material, section 6, for the distribution of random intercepts). Thus, participants differed with respect to the number of refixations they made. This is important because it suggests that differences in recurrence are not simply noise in the data but rather reflect systematic differences between participants, suggesting to different strategies employed by different individuals.

**Figure 6:**
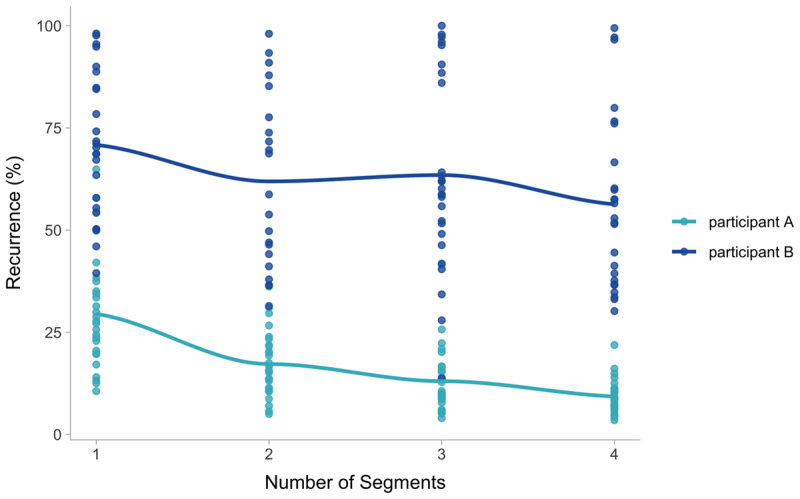
Differences between two participants. Although both show very different degrees of recurrence, the effect of the number of segments is similar. The two participants are chosen to optimally illustrate the statistical finding.

At the same time, the differences between individuals’ general recurrence levels did not influence the way participants adapted their temporal gaze patterns to changing stimuli, given that the correlation between the random intercepts of participants and the random slopes for number of segments was estimated to be around zero in both experiments (Experiment A: –0.29, CI: –0.65 – 0.14; Experiment B: 0.05, CI: –0.38 – 0.49). Thus, regardless of how many refixations participants make, in general fewer refixations are made when the imagined content is more complex. This is illustrated in Figure 6. This means that the processes leading to the reduction of refixations in more complex mental images are the same between participants despite the different styles of eye movements during mental imagery.

**Figure 7:**
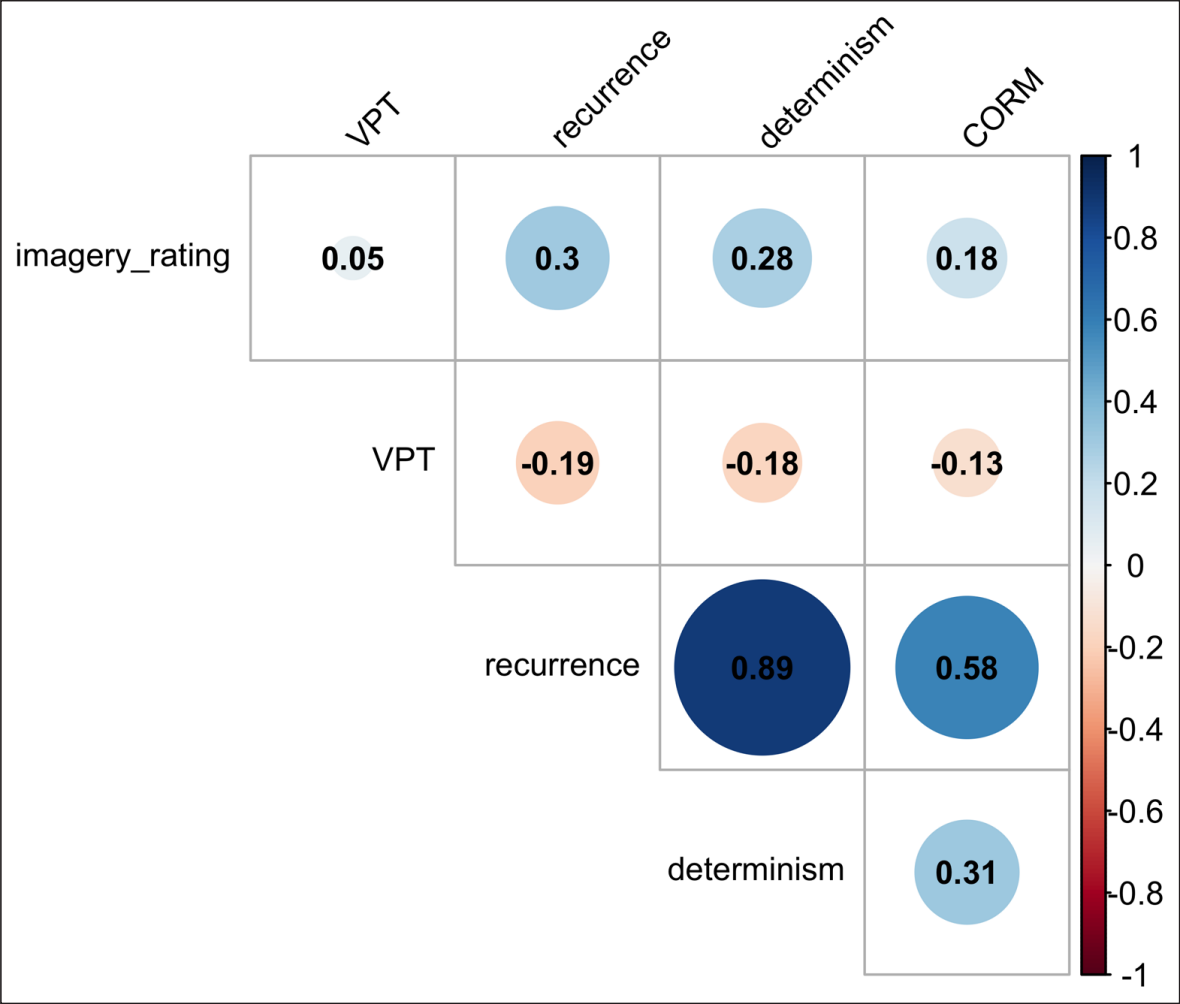
Correlations between measures of temporal gaze dynamics and mental imagery. VPT refers to participants’ score in the Visual Pattern Test. Only the RQA parameters recurrence, determinism and CORM correlate significantly (p < 0.01, adjusted for multiple comparisons).

Turning to the self-report measures, participants also indicated how well they had been able to imagine the stimulus and completed the Visual Pattern Test (VPT) as a measure for short-term memory. Figure 7 shows the correlations between both measures and the temporal gaze dynamics, aggregated over the participants of both experiments. Imagery rating and VPT are uncorrelated. This can be due to the fact that the patterns participants keep in mind in the VPT surpass the complexity of the stimuli they imagined in the maintenance task. It is also possible that the imaginability rating was biased by the answer in the maintenance task that was given immediately before assessing the vividness of the imagined stimulus. Imaginability ratings (i.e. answers to the question “how difficult was it to imagine the stimulus?”) and VPT scores were unrelated to the temporal gaze dynamics, but the temporal gaze dynamics (recurrence, determinism and CORM) are inter-correlated, in accordance with previous literature (Anderson et al., 2013; Zhang, Anderson, & Miller, 2021). The correlation between recurrence and determinism is higher than reported by Zhang et al. (2021), who applied RQA to eye movements in mind wandering during a concurring visual encoding task. This suggests that in mental imagery, recurrent fixations are more systematic compared to when there is concurrent visual input Zhang et al. (2021). This interpretation is confirmed by a re-analysis of the data of Gurtner et al. (2021), where the correlation between recurrence and determinism is significantly higher in mental imagery compared to perception, see Supplementary Material, section 5.2.

## Discussion

We investigated the mechanisms involved in mental image maintenance. Specifically, we proposed that two mechanisms, reactivation of place-bound content and gaze restriction, can be responsible for eye movements during image maintenance. To distinguish between them, we analyzed how temporal gaze dynamics change as the maintenance task becomes more difficult, i.e., participants imagine stimuli increasingly complex segments. Participants were able to complete the maintenance task (average correctness Experiment A: 87.80%, Experiment B: 91.20%) and they complied with the instruction, given that the number of segments influenced their accuracy. The general pattern of eye movement results is the following: when participants maintained a simple stimulus, they made numerous and systematic refixations (recurrence and determinism were both high). Furthermore, spread of fixations was low when the imagined stimulus was simple. As the stimuli became increasingly difficult to maintain, i.e. as the number of segments increased, the spread of fixations increased and participants made fewer and less systematic refixations. This shows that temporal patterns of eye movements change as a function of mental activity associated with imaging complex and non-complex patterns. Interestingly, these results support both reactivation of place-bound content and gaze-restriction.

The analysis of the *spatial* gaze characteristics shows that fixations made when imagining a more complex image were spread out to a greater extent. It is unlikely that this effect is solely due to participants looking at more spread-out segments of the pattern stimulus. First, there were no segments to look at, the screen was blank during the time of eye movement measurement and participants had to maintain the mental image. Second, in principle, there is no one-to-one relationship between complexity and the spread of stimulus elements. Two segments of a simple stimulus can be far apart and four segments in a complex stimulus can be closely arranged (for the relationship between number of segments and the spread of black pixels, see Fig.1 in the Supplementary Material, section 3). Third, we showed that the distribution of the pixels participants had seen did not alter the relationship between the complexity of the imagined stimulus and RQA measures. We argue that as it is more difficult to maintain more complex images, supportive eye movements become necessary. Thus, the spatial gaze characteristics are in line with the idea of ‘looking at nothing’, or reactivation of place-bound content, i.e. the idea that re-fixations can help to counteract fading of a mental image (De Beni et al., 2007; Farah, 1989; Johansson, Holsanova, Dewhurst, & Holmqvist, 2012; Johansson & Johansson, 2014, 2020).

The analysis of the *temporal* gaze dynamics qualifies and extends the results of spatial gaze properties. The spread of eye movements increases with stimulus complexity, which is consistent with reactivation, while the rate of refixations and their systematicity *decrease*. Therefore, refixations cannot result exclusively from reactivation. The origin of refixations becomes more clear when analyzing the relationship between the spread of fixations and recurrence (Figure 5). In principle, high recurrence in mental imagery could originate from widely spread fixations repeatedly targeting the same parts of the mental image. This means, stimuli with more segments could increase the likelihood of refixations, which we hypothesized. Alternatively, high recurrence can originate from closely arranged fixations. Our results show that all trials with high recurrence have a *low* spread of fixations (the median spread of fixations is smaller than the threshold distance between two refixations, see the dotted line in Figure 5). This means that in trials with high recurrence, most fixations are close to each other. High recurrence rates thus originate from keeping the gaze on a restricted area. This pattern cannot be due to the small size of the stimuli used in this study, since the same pattern was present in a re-analysis of previous data based on stimuli covering the entire screen (Gurtner et al., 2021), where stimuli covered the entire screen. Thus, when imagining a simple stimulus, participants actively focus their gaze on a small region, leading to systematic refixations. As the stimulus becomes more complex, additional fixations to stimulus-related areas are needed, and the spread of fixations increases. At the same time, the rate of refixations drops. Thus, we can deduce that these stimulus-related areas are visited only once (if they had been visited repeatedly, spread would increase but recurrence would not drop). Taken together, the analysis of the temporal gaze dynamics suggests that in addition to reactivation of place-bound content (i.e. the ‘looking at nothing’ effect), eye movements in mental imagery also reflect a preference for restricting the gaze on a small area. This conclusion is only possible by analyzing temporal gaze dynamics in addition to summary statistics like the spread of fixations and thus highlights the gains of imagery research when taking into account the temporal dimension of eye movements. The results of the spread of fixations alone would have suggested that ‘looking at nothing’ is the main driver behind eye movements in mental imagery and the importance of gaze restriction would have been overseen.

Gaze restriction makes perceptual information processing less costly, because it implies that over the course of several fixations, the visual input remains rather constant. Constant input, in turn, is more easily suppressed if one wishes to disengage from processing perceptual information and focus on imagined content. Indeed, Craver-Lemley and Reeves (1992) and Reeves and Craver-Lemley (2012) demonstrated that we suppress visual information during visual mental imagery at least when perceptual acuity is high (Reeves, Grayhem, & Craver-Lemley, 2020), and there is evidence that the feedback circles of perceptual inference change during mental imagery (Dijkstra, Ambrogioni, Vidaurre, & Gerven, 2020). This disengagement might be reflected in the restriction of the gaze to a small area, which could help the maintenance of mental images. Accordingly, there is growing evidence that eye movements in mental imagery can interfere with mental imagery (J. Pearson et al., 2008; Teng & Kravitz, 2019; Wais et al., 2010). Exactly how, when, why, and to what degree this disengagement from analyzing visual content during mental imagery takes place is an interesting and important avenue for future research.

We suggest that the complexity of the mental image is an important factor that determines the relative importance of gaze restriction vs. making place-bound eye movements to support mental imagery. Restricting gaze might enable participants to imagine the stimulus more holistically as long as the stimulus’ complexity allows for this strategy. As the stimulus becomes more complex, this strategy might not be feasible because too much information would fall outside of the central part of the mental image. In this case, making eye movements to update the content in the periphery might enable maintenance of the entire mental image. This interpretation is supported by the fact that, as complexity increases, participants make more spread out, and more unique eye movements (lower recurrence and lower determinism). It follows that fixations in mental imagery can reflect reactivation of place-bound content but that they do not do so in every case. Eye movements in mental imagery are thus shaped by another process beyond the ‘looking at nothing’ effect (Chiquet, Martarelli, & Mast, 2020). High rates of refixations can be driven by active disengagement from perceptual information processing. The effect of the complexity on recurrence was controlled for the spread of fixations (Gurtner et al., 2019, 2021) and our results highlight processes that have been neglected in the study of eye movements in mental imagery.

The overall effect of image complexity on temporal gaze dynamics was accompanied by large differences between participants in the number of refixations they made. This is in accordance with previous findings about the temporal dynamics of eye movements during mental imagery (Gurtner et al., 2021). Interestingly, Kinjo, Fooken, and Spering (2020) showed that a third of their participants were “non-saccaders”, as they made few saccades during retrieval. The authors interpreted this as an individual preference in the trade-off between the benefit and the cost of making eye movements. At the same time, both “saccaders” and “non-saccaders” showed comparable memory performance. Likewise, our participants were able to solve the maintenance task despite considerable interindividual variance of the number and systematicity of refixations. It is possible that the individual cost and benefit of eye movements in mental imagery (Kinjo et al., 2020) can explain the variance in temporal gaze dynamics between participants.

This variance can be due to individual representation styles. Palmiero et al. (2019) suggest that individual combinations of imagery ability and strategy (i.e. object and spatial imagery, Bochynska & Laeng, 2015) lead to different forms of mental representations. Indeed, the type of mental representation could be the cause of differences in eye movements between learning styles (Cao & Nishihara, 2012) and it might be associated with the differences in eye movements that accompany navigation styles, i.e. whether an individual memorizes a map by landmarks, the route they have to remember or in a survey-style (Piccardi et al., 2016). The different types of mental representations could further produce the discrepancies in temporal gaze dynamics between experts and novices (Gandomkar, Tay, Brennan, & Mello-Thoms, 2017; Vaidyanathan et al., 2014).

Likewise, the representation participants base their mental images upon could influence temporal gaze dynamics in mental imagery. Participants with a non-spatial representation-style, i.e. object-imagers (Bochynska & Laeng, 2015), show diminished ‘looking at nothing’ (Chiquet, Martarelli, & Mast, 2022) and could be those who do not move their eyes much during imagery (non-saccaders, Kinjo et al., 2020). In their case, planning, executing and verifying eye movements could add noise, while their representation would not gain much from making stimulus-related eye movements. This interpretation is in line with our previous finding that working memory modulates the ‘looking at nothing’ effect (Gurtner et al., 2021). Larger working memory capacity leads to a spatially more accurate mental image, which, in turn, increases the spatial correspondence with the perceptual scenario. There might thus be different forms of mental representations associated with different eye movement dynamics in mental imagery. At the same time, the mechanism by which image complexity influences eye movement dynamics appears to be the same for everyone since individual recurrence levels did not influence the effect of complexity on eye movement dynamics.

Despite our efforts to maximize the interfering potential and to our surprise, the DVN in Experiment B did not influence the temporal gaze dynamics, the spread of fixations, or the accuracy in the maintenance task. The mental images were robust and did not fade more rapidly during the exposure to DVN. One possible reason for this is the type of stimuli we used. It is possible that participants maintained the geometric stimuli in the visual cache, which is more protected from incoming sensory data (J. Pearson & Keogh, 2019), instead of holding them in the visual buffer (Borst et al., 2006; Cocchini, Logie, Della Sala, MacPherson, & Baddeley, 2002) where the DVN might have a stronger effect. Furthermore, the influence of DVN on mental imagery can be particularly strong when visual details of the mental image were tested (Dean et al., 2005, 2008; Dent, 2010; McConnell & Quinn, 2004; Orme, 2009). Our stimuli might not have contained enough visual details for the effect of DVN to unfold. Finally, our task is similar to classical short-term memory tasks, and we thus used non-structured DVN. However, structured DVN might disrupt the mental images more efficiently. Structured noise has been used by Borst, Ganis, Thompson, and Kosslyn (2012), but their task differed substantially in timing (1.5 sec of image generation and maintenance vs. 20 sec of maintenance in our study) and the specific mental operation (image inspection vs. spatial judgment in our study). The task used by Dror and Kosslyn (1994) has several advantages for the aim of our study. First, it is based on the theoretical model of mental imagery by Kosslyn (1994) and it was designed specifically to test the image maintenance phase (separating it from image generation and inspection). Second, the task accounts for the fact that we group several black cells into a single segment (i.e. four black cells in a row result in a complexity of one, not four), which is unlike similar tasks used in the context of DVN. Vasques, Garcia, and Galera (2016) used the number of black cells but re-analysis of our data with their definition did not change the overall results substantially. Third, unlike more naturalistic pictures, the degree of picture complexity is controllable in a systematic fashion. Nevertheless, future research could consider other measures of stimulus complexity, such as entropy, especially with regard to natural pictures in which scene complexity cannot be varied easily. Beyond such methodological considerations, one could also speculate that the lack of an effect of DVN on gaze dynamics suggests that gaze properties are independent of short-term memory processes. Such speculation would also be consistent with the low correlation of the VPT with gaze dynamic measures, but warrants more research in the future.

Our study has two possible limitations. Participants could have restricted their gaze because they were imagining a comparatively small stimulus. However, we re-analyzed the data of a previous paper (Gurtner et al., 2021), where participants imagined scenes that covered the entire screen. In that study, the relationship between spread and recurrence is comparable to the non-linear relationship reported here. Therefore we conclude that temporal gaze dynamics are not a simple function of the size of the stimulus.

In addition, the definition of recurrent fixations depends on the choice of a threshold distance between fixations. The threshold is often defined so that it matches the size of the fovea, which is meaningful in the case of perception. It is not clear in how far this convention is suitable for mental images. We therefore analyzed our data with different threshold definitions (see Supplementary Material, section 7). As expected, the relationship between spread and recurrence changes but still, no participant made many refixations at the same time as moving their eyes over large distances. Thus, altered thresholds do not entail substantial changes in the results.

## Conclusion

As the complexity of mental images increased, participants made fewer and less systematic refixations, while the spread of fixations increased. We show that refixations in mental imagery originate, at least partly, from a tendency to restrict the gaze, and this reflects disengagement from perception. In addition, simple mental images can be maintained without making eye movements, whereas complex mental images require stimulus-related fixations. The results suggest that eye movements in mental imagery are shaped by two mechanisms: the ‘looking at nothing’ effect to reactivate part-based pictorial content and the minimization of interference from unrelated perceptual input. The trade-off between the cost and the benefit of making eye movements could cause the pronounced interindividual differences in temporal gaze dynamics.

## Data Accessibility Statement

The data will be made available at the Open Science Framework. To access the study materials and the analysis code, please contact the corresponding author (Lilla Gurtner). This study was not preregistered.

## Additional File

The additional file for this article can be found as follows:

10.5334/joc.316.s1 Supplementary Material. The supplementary material consists of additional analysis to the results presented in the main article.

## Footnotes

Both approaches compute scan path similarity. ScanMatch separates the screen into small quadrants and compares how similar the sequences of quadrants are, in which participants made fixations. MultiMatch converts the scan path into a vector representation, which allows to compare the scan path shape independently from its size, for example.
