# Supplementary material for: Gaze Restriction and Reactivation of Place-bound Content Drive Eye Movements During Mental Imagery
Source: J Cogn. 2023 Aug 31;6(1):51. doi: 10.5334/joc.316 (PMC10473167; doi:10.5334/joc.316)
Supplement: Supplementary Material. — The supplementary material consists of additional analysis to the results presented in the main article. [file joc-6-1-316-s1.pdf]

Supplementary Material: Gaze restriction and reactivation of place-bound content drive eye movements in mental imagery

Lilla M. Gurtner[1], Walter F. Bischof[2], and & Fred W. Mast[1]

[1] Department of Psychology

University of Bern

Bern

Switzerland

[2] Department of Psychology

University of British Columbia

Vancouver BC

Canada

Author Note

The authors made the following contributions. Lilla M. Gurtner: Conceptualization, Data curation, Formal analysis, Methodology, Project administration, Visualization, Writing - Original Draft Preparation, Writing - Review & Editing; Walter F. Bischof: Conceptualization, Writing - Review & Editing; Fred W. Mast: Conceptualization, Supervision, Writing - Review & Editing.

Correspondence concerning this article should be addressed to Lilla M. Gurtner, Fabrikstrasse 8, 3012 Bern, Switzerland. E-mail: lilla.gurtner2@psy.unibe.ch

Supplementary Material: Gaze restriction and reactivation of place-bound content drive eye movements in mental imagery

The Supplementary Material contains additional information with regard to the variables and analyses.

1. Model specifications

We used the brms-package (Bürkner, 2018) to fit our models to the data. Here, we provide the formulae used to fit the different models.

RQA fits

Experiment A:

```
RQA measure / 100 ~ 1  + nSegments * spread +
               (1 | stim_name) + (1 + nSegments * spread | participant)
```

Experiment B:

```
RQA measure / 100 ~ 1  + nSegments * spread + DVN +
               (1 | stim_name) + (1 + nSegments * spread + DVN | participant)
```

Spread of fixations

Experiment A:

```
spread of fixations ~ 1  + nSegments +
               (1 | stim_name) + (1 + nSegments | participant)
```

Experiment B:

```
spread of fixations ~ 1  +nSegments * DVN +
```

```
(1 | stim_name) + (1 + nSegments * DVN| participant)
```

2. Deletion of trials

We excluded trials with low imaginability ratings (equal or below 2) from our analysis to ensure that we only analyze trails in which imagery was successful. Furthermore, we excluded trials in which participants gave wrong answers. Since the stimuli increased in difficulty, this could potentially lead to more deletions of stimuli with more segments. However, this was not the case, see table 1.

Table 1

Deleted trials

experiment	n of segments	n deleted trials
A	1	18
A	2	24
A	3	19
A	4	13
B	1	24
B	2	26
B	3	23
B	4	28

Note. Shows the number of trials that were deleted because of wrong answers or imaginability ratings equal to or lower than 2.

3. Spread of black pixels

The spread of the stimuli might be a potential confound of the effect of stimulus complexity on recurrence. We address this issue twofold, with a mediation analysis and by means of model comparisons. Third, we explore the effect that the spread of pixels has on the spread of participants' fixations.

We computed the spread of black pixels in each stimulus in an analogous fashion to the spread of fixations: First, we computed the mean of the x and y locations of all non-white pixels. Then, we computed distance of all non-white pixels to this average location. The mean of all these distances represents the spread of black pixels. Figure 1, illustrates the relationship between the spread of black pixels and the number of segments.

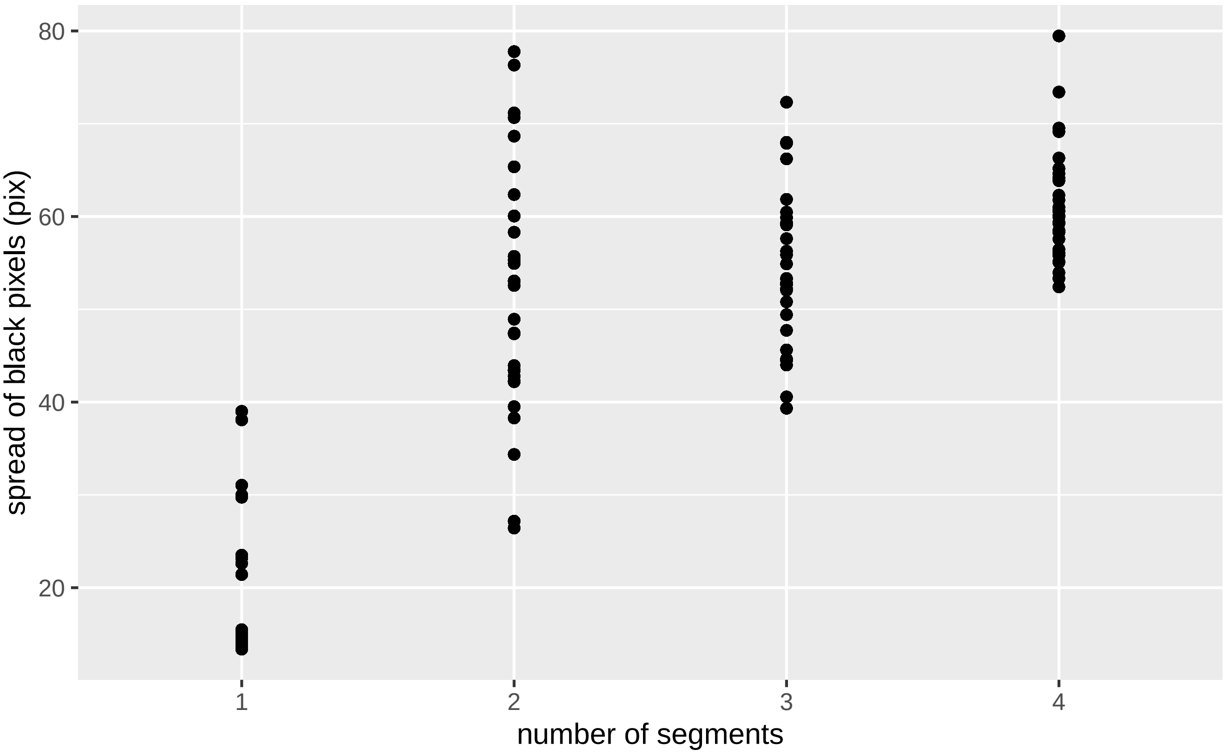

Figure 1

The relationship between the spread of black pixels and the number of segments.

We tested, whether the effect of the number of segments on recurrence is mediated by

the spread of black pixels in the stimuli participants maintained while the screen was blank. An effect of the pixel spread of an absent stimulus on gaze behavior is plausible if we assume a strong ‘looking at nothing’ effect (that is, gaze behavior is predominantly determined by the original stimulus as presented). To test whether the pixel spread is responsible for the effect of the number of segments on recurrence, we conducted a mediation analysis (following Kurz, 2019). We used a multivariate regression model in which we defined the two models as follows:

```
model_a: recurrence ~ 1 + spread + nSegments + pixel_spread
model_b: pixel_spread ~ 1 + nSegments
```

We used a lognormal link function for model_a (a zero-one-inflated beta link function did not result in a satisfying overlap between predicted and actual recurrence values), and a student_t link function for model_b. The distribution of pixel spread was bimodal, with one mode for all one-segment stimuli consisting of just one black cell. Since this was very hard to fit with any link function, we decided to filter out all trials with a average pixel spread lower than 20. Unfortunately, we could not include random effects since the chains did not mix well under these circumstances. The mediation analysis showed that there is no indirect effect of the number of segments on recurrence. The posterior distribution of the indirect effect is shown in Figure 2. Thus, we conclude that there is no evidence for a mediation effect.

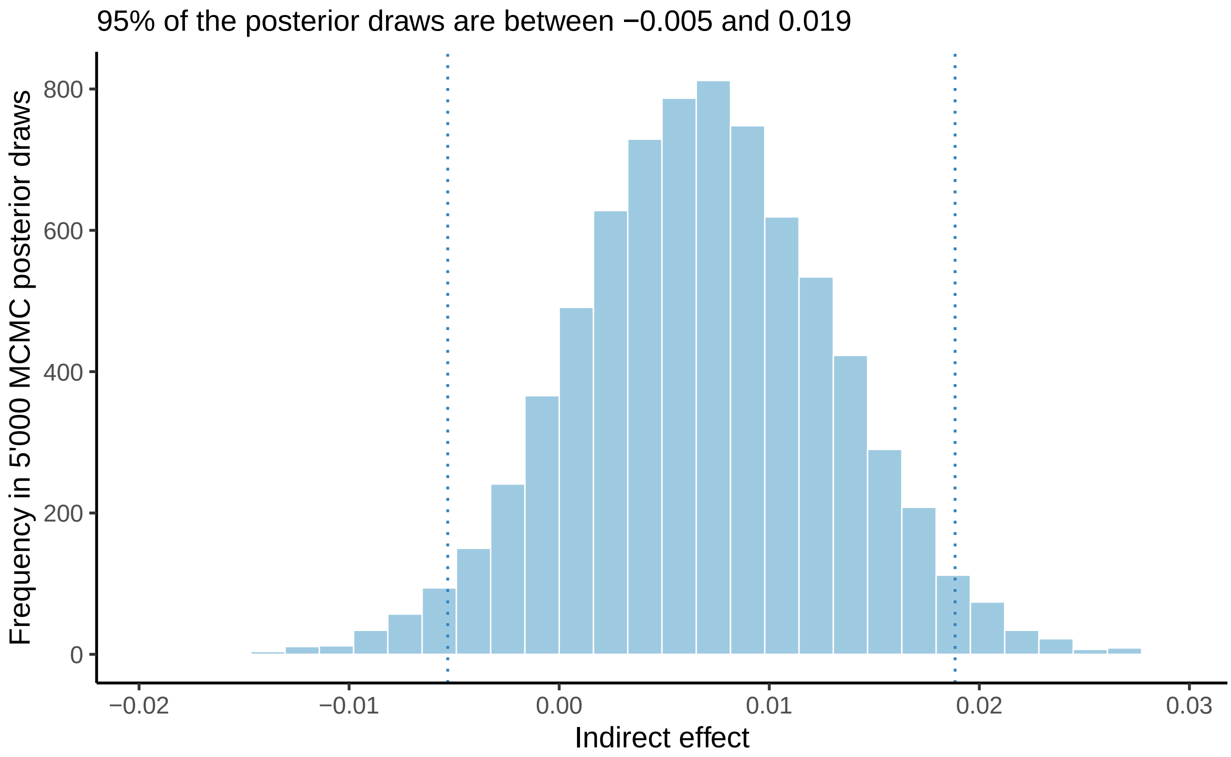

Figure 2

Posterior distribution of the indirect effect of number of segments on recurrence, taking into account the possible mediation by pixel spread. Since the distribution clearly includes zero in its 95% credibility interval, we conclude that there is no evidence for a mediation effect.

The mediation analysis we performed lacked multi-level structure and we did not include all data in it. Hence, we confirmed its results by means of a model comparisons approach.

In the model comparison approach, we compared two regression models, one of which contained the pixel spread as predictor of gaze behavior and one of which did not. We tested, whether adding pixel spread as predictor altered the effects of the other predictors and whether the more complex model gave a better model fit, i.e. a better description of the data.

The pixel spread did not predict recurrence values in both experiments (Experiment

A: beta = 0.00, CI: 0.00 - 0.00, Experiment B: beta = 0.00, CI: 0.00 - 0.00). Including pixel spread did not alter the effect of the number of segments substantially as can be seen in the difference between posterior distributions of the effect in the two respective models (Experiment A: median of difference between posterior distributions = 0.00, CI: -0.16 - 0.18, Experiment B: median = -0.02, CI: -0.15 - 0.12). Finally, the model comparison showed that the models without the distribution of the black pixels outperformed those that contained the black pixels' distribution as a predictor (difference of expected log predictive densities (elpd) from Experiment A: -1.47, se_diff = 0.65, Experiment B: -1.69, se_diff = 1.63).

Finally, the spread of the pixels in the stimulus might predict the spread of fixations, assuming a strong 'looking at nothing' effect. We tested this with a lognormal regression using the formula:

```
spread_of_fixations ~ 1 + pixel_spread * nSegments + Experiment +
                          (1 + pixel_spread * nSegments|vp) +
                          (1 + pixel_spread*nSegments | stim_name)
```

We found that the spread of pixels did not predict the spread of fixations (beta coefficient: 0.00, lower CI: 0.00, upper CI: 0.01. Taken together, these results make us confident that stimulus complexity in fact influenced gaze behavior and is not confounded with the spread of pixels in the maintained stimulus.

4. Effects of DVN

Dynamic visual noise did not influence the recurrence measures or the spread of fixations. In table 2, we present the estimated beta coefficients for the DVN in all four models. Again, we compared models in order to show that adding DVN does not increase the predictive power of the respective models. The results of the model comparisons are shown in table 3. DVN had no effect on gaze properties, suggesting that any potential retinocentric afterimages did not systematically influence participants' gaze behavior, as the

Table 2

Effect of DVN on eye movements

dependent variable	beta coefficient	CI_{lower}	CI_{upper}
recurrence	-0.01	-0.06	0.05
determinism	-0.01	-0.07	0.06
CORM	-0.01	-0.04	0.02
spread	-1.85	-6.81	3.01

Note. Evidence for the absence of an effect of the dynamic visual noise on recurrence parameters and on the spread of fixations.

gaze properties remained the same regardless of whether afterimages were masked with DVN or not.

Table 3

Predictive power of dynamic visual noise

dependent variable	elpd difference	se(elpd)
recurrence	-2.22	0.64
determinism	-2.13	0.80
CORM	-1.04	1.89
spread	-20.01	9.03

Note. The difference of expected log predictive densities (elpd) of models that contain DVN as a predictor and models that do not. Negative values indicate that in all cases, the model that contains DVN has lower predictive power.

5. Reanalysis of previous data sets

5.1 Relationship between recurrence and spread

The present experiment shows a specific, non-linear relationship between the spread of fixations and recurrence. It could be argued that the relationship between recurrence and the spread of fixations is caused by the specific spatial layout of our stimuli. We therefore reanalyzed data from a recent publication (Gurtner, Hartmann, & Mast, 2021). In this experiment, the stimuli (art, faces and landscapes) covered the entire screen. Figure 3 shows that the pattern of the relationship is similar to the one presented in the main article.

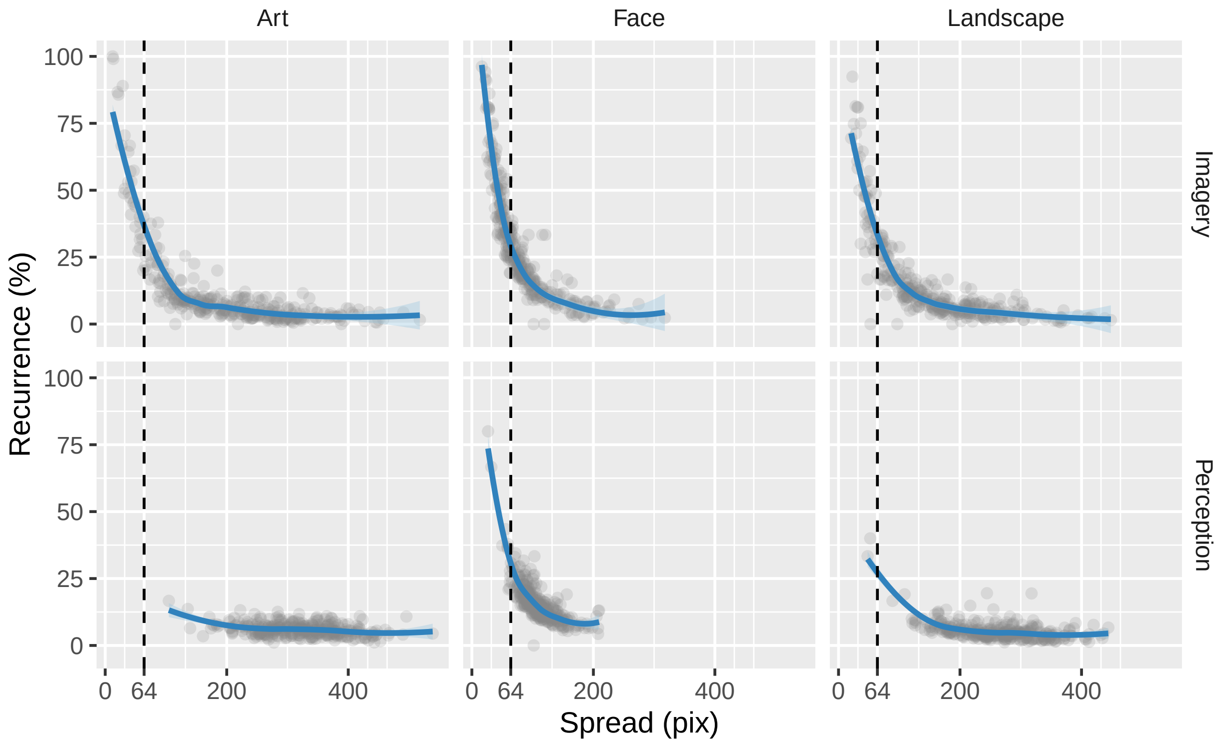

Figure 3

Relationship between the spread of fixations and recurrence in stimuli that covered the entire screen. The pattern of the relationship in mental imagery is strikingly similar to the pattern in the main article.

5.2 Correlation between recurrence and determinim

Recurrence and determinism of eye movements are correlated in perception (Anderson, Bischof, Laidlaw, Risko, & Kingstone, 2013) and also during mind wandering (Zhang, 2020). In mental imagery, the correlation between recurrence and determinism was particularly high (0.89 compared to 0.67 in Zhang (2020) for example). To test whether the correlation in mental imagery was significantly higher compared to perception, we reanalyzed data from a recent publication (Gurtner et al., 2021), where participants saw a picture (art, face or landscape) and subsequently imagined it for 15 seconds each. The correlations between recurrence and determinism are not independent in this case, since we used repeated measures over participants. Therefore, standard methods to compare two correlation coefficients were not applicable. Instead, we performed a permutation test on the difference between the two correlations. Specifically, we tested whether the difference between the correlation in perception and mental imagery was higher than what could be expected by chance. This was the case in 96.53% of the cases, corresponding to a p-value of 0.03. Thus, in mental imagery, the association of recurrence and determinism is higher than in perception. This means that in imagery, more of refixations are part of systematic refixation patterns when compared to perception.

6. Distribution of random intercepts

Multi-level models allow for assessing inter-individual variance by estimating random intercepts. We use this possibility to further illustrate that the large observed over-all variance in RQA parameters is caused by consistent inter-individual differences in RQA values. The random intercepts for each person are distinct from each other and not include zero in their CIs (see Figure 4). This means that participants differ from the overall recurrence level in idiosyncratic ways, supporting the notion of large interindividual differences in eye movements during imagery.

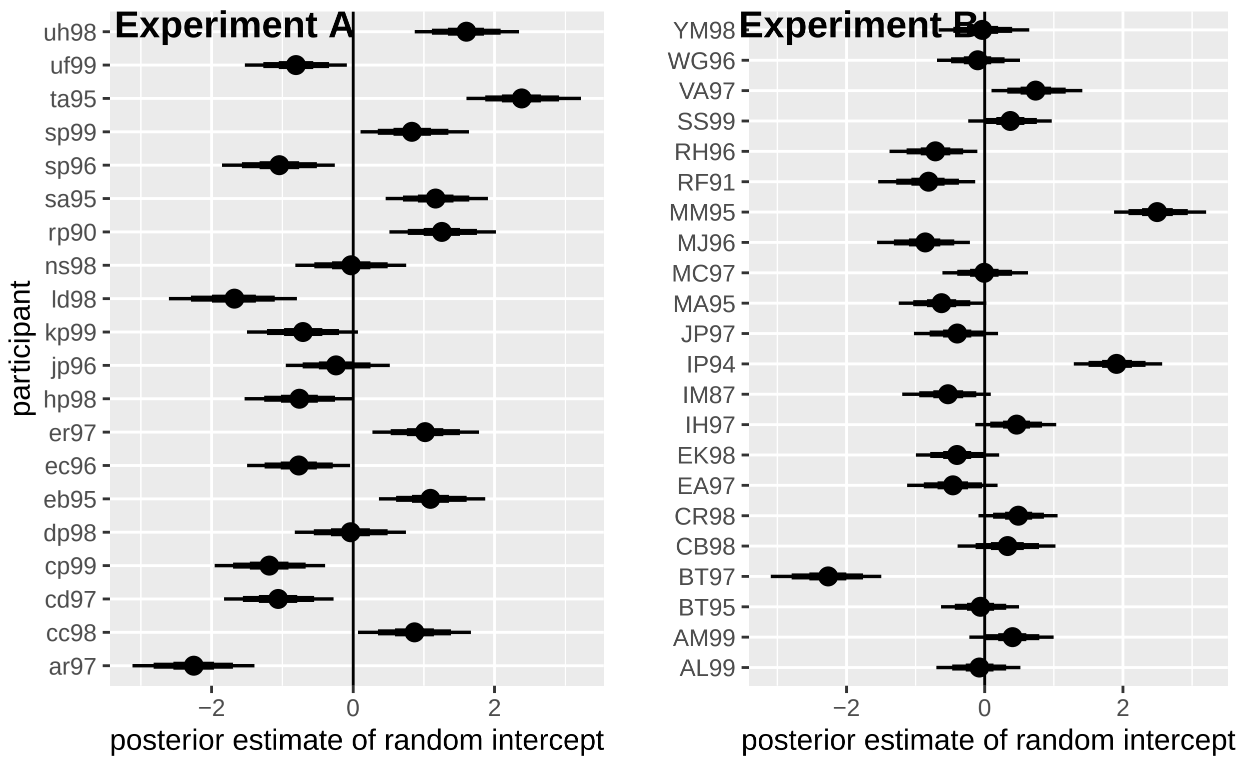

Figure 4

Posterior estimates of the random intercepts for each participant in both experiments.

7. Recurrence thresholds

The calculation of recurrence depends on the choice of the threshold distance below which two fixations are considered recurrent. Often, the threshold is chosen as the diameter of the fovea. In the case of mental imagery, it is questionable, whether this choice of a threshold is justified. We therefore re-analyzed the data with different thresholds. Figure 5 shows how the relationship between the spread of fixations and recurrence changes, as the threshold for defining refixations increases. The stimulus we used measured 462 x 567 pixels, each cell within the stimulus measured 109 x 108 pixels, the screen measured 1280 x 1024. This information can provide a reference for the threshold choices. Note that, in order to see high recurrence at the same time as very widely spread out fixations, the threshold to define recurrence must be set to be almost the size of the entire screen, at which point RQA becomes meaningless as we excluded fixations out of the screen.

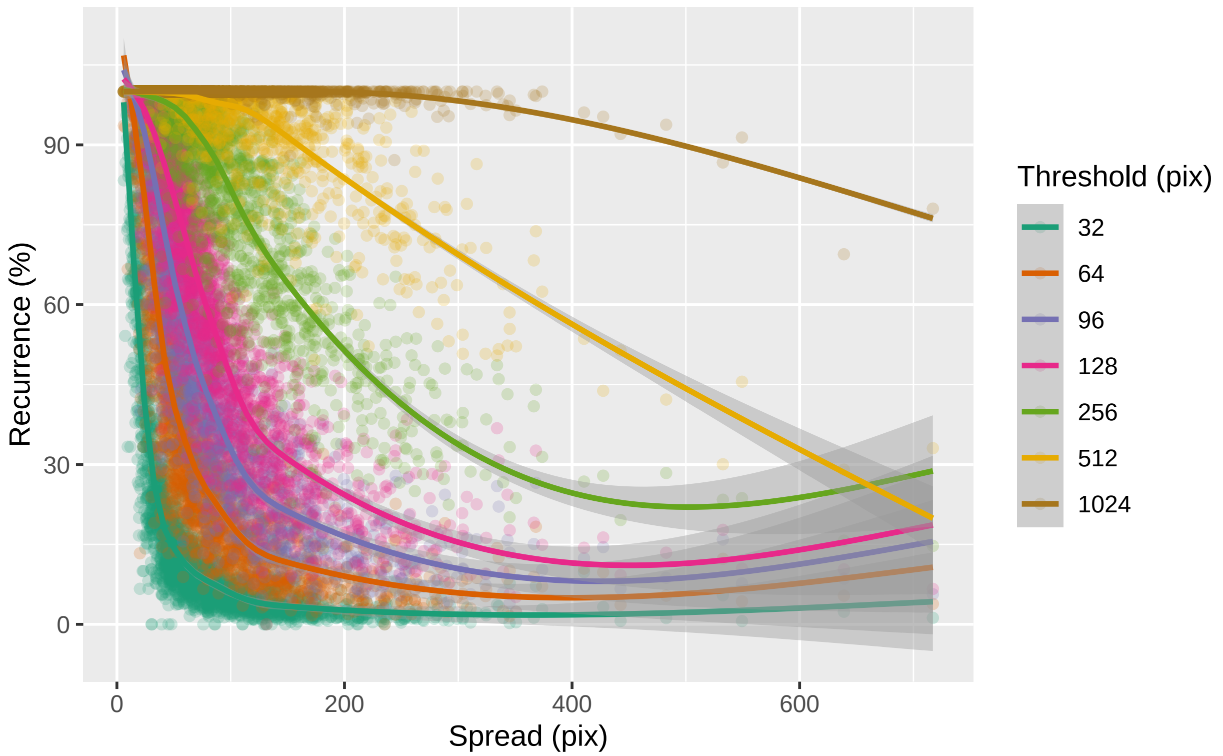

Figure 5

The relationship between recurrence and spread of fixations as the definition for refixations change.

To test whether the effect of the stimulus complexity on temporal gaze dynamics depended on the choice for the thresholds, we reran the analysis of the main article with different criteria to define recurrence. This was done for the threshold distances of 32, 96, 128, and 256 pixel. Figure 6 shows how the effect of stimulus complexity on recurrence changes with the choice of the threshold distance. As the threshold distance between recurrent fixations increases, the effect of the complexity on recurrence vanishes (the posterior samples are centered around zero). At the largest threshold definition, most fixations are considered recurrent and no variance between the stimuli is left and the effect of stimuli complexity on recurrence vanishes.

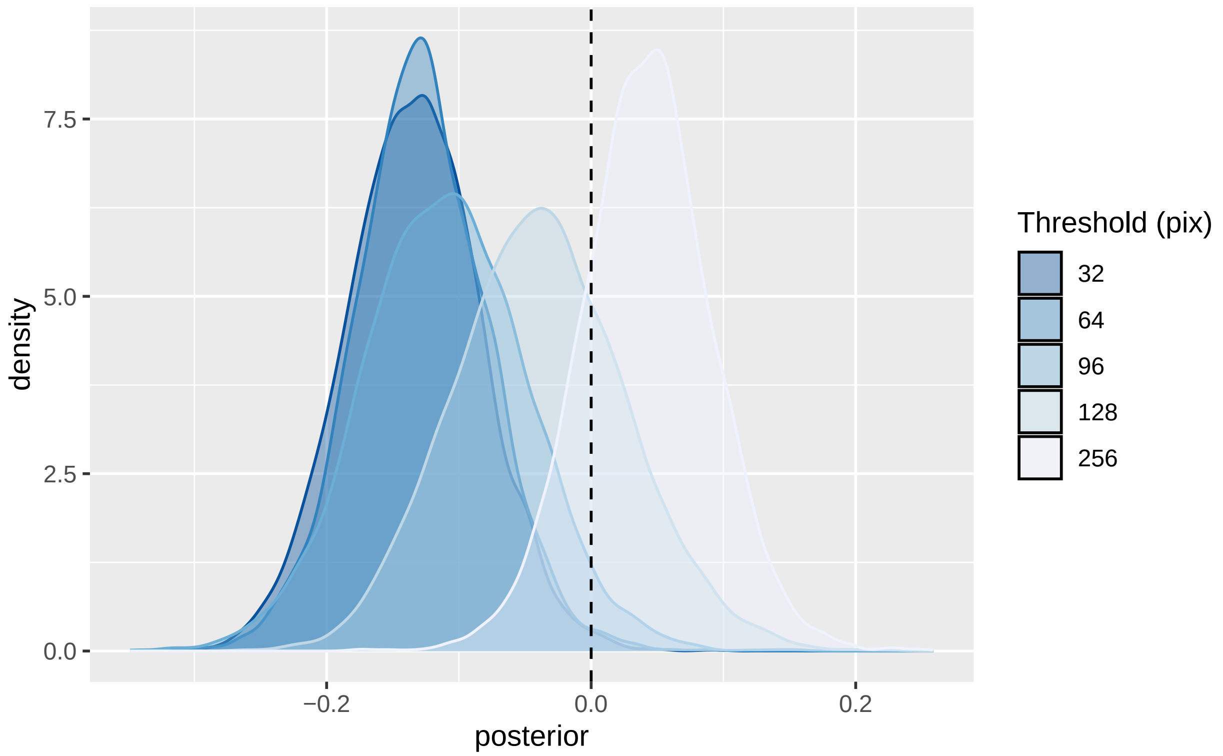

Figure 6

Posterior samples of the effect of complexity on recurrence, depending on the choice of the threshold distance between two recurrent fixations. The analysis of the main article used a threshold distance of 64 pixel, approximately the size of the fovea.

8. Imaginability ratings and complexity

The number of segments in the stimuli influenced participants' performance (Figure 5 in the article). However, their subjective experience ratings did not show a similar effect (see Figure 7). The mean rating on the y axis indicates how well participants were able to imagine the stimuli on a scale from 1-7. On average, participants gave high ratings for all complexity categories, which is in line with the ceiling-effect in performance we report in the manuscript in Figure 5 in the main article. Hence, the task was easy objectively and subjectively. Nevertheless, the objective performance assessment was able to show a decrease in higher complexity stimuli, but this apparently did not translate to the subjective experience of participants (no decrease in Figure 7 below). The relationship in Figure 7 is

independent of how we operationalize picture complexity. It looks similar if we operationalize “stimulus complexity” as the number of cells as indicator for complexity or by multiplying the number of cells with the number of segments. Therefore, we have decided to refrain from conducting further analysis in this regard.

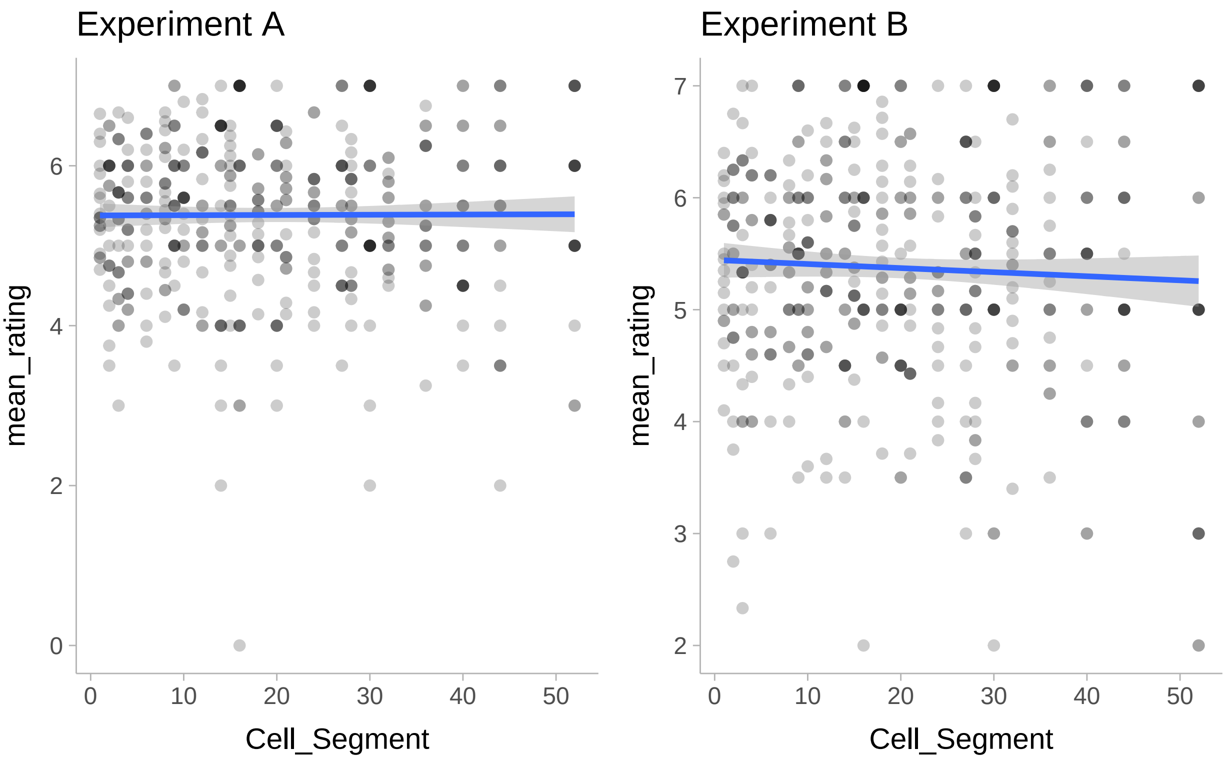

Figure 7

Relationship between the spread of fixations and recurrence in stimuli that covered the entire screen. The pattern of the relationship in mental imagery is strikingly similar to the pattern in the main article.

References

Anderson, N. C., Bischof, W. F., Laidlaw, K. E. W., Risko, E. F., & Kingstone, A. (2013). Recurrence quantification analysis of eyemovements. *Behavior Research Methods*, *45*, 842–856. https://doi.org/10.3758/s13428-012-0299-5

Bürkner, P.-C. (2018). Advanced Bayesian multilevel modeling with the R package brms. *The R Journal*, *10*(1), 395–411.

Gurtner, L. M., Hartmann, M., & Mast, F. W. (2021). Eye movements during visual imagery and perception show spatial correspondence but have unique temporal signatures. *Cognition*, *210*, 104597. https://doi.org/10.1016/j.cognition.2021.104597

Kurz, S. (2019). *The simple mediation model.* Retrieved from https://bookdown.org/ajkurz/recoding_Hayes_2018/the-simple-mediation-model.html

Zhang, H. (2020). *Mind-Wandering: What Can We Learn from Eye Movements?* https://doi.org/10.31234/osf.io/n9fbz
